# Use of a Glaciogene Marine Clay (Ilulissat, Greenland) in a Pilot Production of Red Bricks

**DOI:** 10.3390/ma17174365

**Published:** 2024-09-03

**Authors:** Louise J. Belmonte, Lisbeth M. Ottosen, Gunvor M. Kirkelund

**Affiliations:** Department of Environmental and Resource Engineering, Technical University of Denmark (DTU), 2800 Lyngby, Denmarklimo@dtu.dk (L.M.O.)

**Keywords:** Arctic, marine sediment, construction material, fired brick, brickwork, pilot

## Abstract

Uplifted occurrences of fine-grained glaciogene marine sediments are found throughout the northern hemisphere. These sediments could be used to produce local construction materials, to rely less on imported construction materials from southern regions. In this study, a representative occurrence from Ilulissat, West Greenland, was investigated as a potential resource for local brick production. The study comprised three parts: (1) raw material characterization based on grain size distribution, major element chemistry, including total carbon, sulfur, and chloride concentrations, mineralogy, morphology, and Atterberg limits; (2) the production of test bricks at a Danish brickwork; and (3) testing of the bricks based on total shrinkage, water absorption, hygroscopic adsorption, open porosity, bulk density, compression strength, and mineralogy. The bricks produced proved to have excellent compression strength, low open porosity, and low water absorption. The shrinkage could be reduced by adding 10% chamotte to the marine sediment. Based on the investigated properties, this indicates that this type of clay is highly suitable as a resource for bricks.

## 1. Introduction

The Arctic climate places high demands on the construction materials used, which are, at the same time, mostly imported from southern regions, in the climatic conditions in which they were intentionally developed. The high cost and logistical challenges of importing construction materials to the Arctic make it relevant to explore locally sourced materials. Large occurrences of fine-grained glaciogene marine sediments were deposited after the last glaciation and some later uplifted above sea level due to isostatic movements. Today, they can be found all over the formerly glaciated northern hemisphere regions, such as Canada, northern Scandinavia, and Greenland [1,2,3,4,5,6]. The marine sediments are dominated by a <60 µm fraction (clay and silt fractions) and contain only a minor amount of the 60–2000 µm fraction (sand fraction).

Studies of Greenlandic, Norwegian, and North American marine sediments have shown qualitatively similar mineralogy results often consisting of feldspars, quartz, amphibole, mica/illite, chlorite, expandable clay minerals (e.g., smectite, vermiculite, and mixed layer clays), and calcite, and their fine and colloid fractions contain a high percentage of primary minerals, such as quartz, feldspars, and amphibole [1,2,3,4,5,7,8,9]. The chemical composition of the studied samples of marine sediments from this region is shown in Table 1, compared to marine sediments from other areas and Danish clay, both used for brick production.

In West Greenland, quaternary glaciogene marine sediment deposits are observed throughout the inhabited coastal regions and are, in most cases, easily accessible from towns and settlements. In addition, the Greenlandic ice sheet is continuously transporting sediments toward the coastal areas for deposition [18], which make sediments an abundant resource in Greenland. The geology of West Greenland is dominated by Precambrian crystalline basement rocks (approximately 3.8–1.7 Ga) consisting of gneisses (associated with the greenschist to granulite facies metamorphism) and some mafic, ultramafic, pegmatitic, and granitic occurrences [19]. Larger occurrences of quaternary marine sediments have also been mapped in these areas [20], and the chemical composition of the sediments will vary to some extent based on their provenance area. The Greenlandic marine sediments in Table 1 include samples from the Nagssugtoqidian orogen [21] (Ilulissat, Kangerlussuaq, and Qasigiannguit) as well as the North Atlantic Craton (Ilulialik). The Greenlandic sediments generally fall within the same range of chemical composition as Canadian sediments. However, in comparison to Norwegian sediments, Greenlandic sediments contain slightly more SiO_2_ and Na_2_O and slightly less Fe_2_O_3_T (i.e., total Fe_2_O_3_), MgO, and K_2_O. This reflects regional differences in sediment provenance.

Research has established that marine sediments are suitable as primary and secondary resources in the production of clay ceramics [8,9,10,11]. The chemical composition of Greenlandic marine sediments is overall comparable to the chemical composition of marine sediments [13,14,15,16] and Danish clay [17] that have previously been used for bricks (Table 1). Today, bricks are neither commonly used nor produced in Greenland, where construction materials, such as wood and concrete, are dominant [22], and the total import of materials for building and construction is about 25% of the total import to the country [23]. Depending on the raw materials and production methods, bricks have several advantages that make them an interesting building material in Arctic climate regions, such as Greenland. They are durable and require almost no maintenance compared to, e.g., wood. Furthermore, they are fire-resistant and have good thermal and acoustic properties [24]. Previous studies of small clay discs (d = 20 mm, h = 3 mm) produced using Greenlandic marine sediment, combined with local particulate waste materials, such as waste incineration ashes and mine tailings, have shown physical and chemical properties that would make the marine sediment suitable for brick production [10,25]. Brick discs with Greenlandic marine clay from the town of Sisimiut even showed less shrinkage during firing, as well as lower porosity and water adsorption and a higher density than brick discs made from commercially used Danish clay material [10,25]. Low shrinkage and high density indicate that Greenlandic marine sediments will be stable during brick production and result in strong bricks, whereas low porosity and water absorption can indicate a more durable brick. Therefore, there could be potential in producing bricks from Greenlandic clay materials, and these promising laboratory-scale results are to be verified by real-size bricks produced in industrial settings. In this study, marine sediment from the town of Ilulissat, West Greenland, was characterized and tested for use in industrial brick manufacturing. Furthermore, the technical suitability of the produced bricks as a local building material was evaluated by parameters such as compression strength and water absorption.

## 2. Materials and Methods

### 2.1. Materials and Sampling

**Figure 1:**
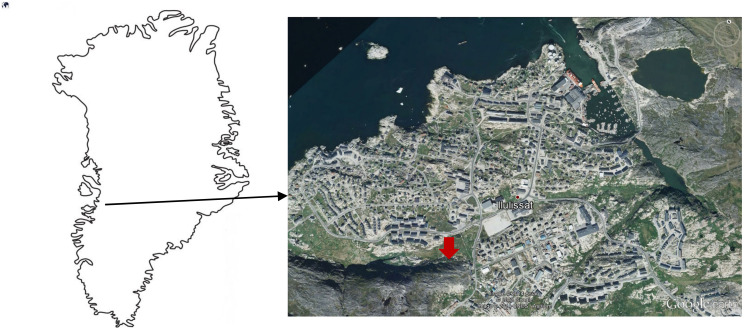
Location of sample extraction (marked by the red arrow): Ilulissat, Greenland.

The investigated raw material was a fine-grained glaciogene marine sediment (MS) from the town of Ilulissat, West Greenland (sampling point red arrow (22 W 0495779 7678250) in Figure 1). Approximately 150 kg of MS was sampled from a depth interval of 20–100 cm below surface. The material was roughly homogenized with a shovel and distributed into 10 L plastic buckets, which were sealed and stored at room temperature until use. Furthermore, two additional raw materials (CR and CH) were used to produce the bricks. CR is a fine-grained residue produced from crushing granitic rocks for aggregate production, which is produced at Betoncentralen, Nuuk, Greenland. CH—chamotte—is a ceramic product with a high alumina and silicon content. CH is currently not produced in Greenland, but can be manufactured by crushing and milling discarded bricks from a future production.

### 2.2. Characterization of Raw Material

The particle size distribution of MS, CR, and CH was determined by laser diffractometry. The samples were pre-dispersed in approximately 20 mL 0.005 M Na_4_P_2_O_7_ using ultrasonics, and then analyzed by wet dispersion in deionized water on a Malvern Mastersizer 2000 laser diffractometer (Malvern Instruments, Malvern, UK). The Mie theory was applied for calculating the particle size distribution using a real refractive index of 1.5 and an imaginary refractive index of 0.1.

Majorbelement analyses were determined using the X-ray fluorescence (XRF) technique on pressed powder tablets prepared from crushed bulk material. The tablets were analyzed on a PanAlytical MagiX PRO (PanAlytical, Almelo, The Netherlands) equipped with a Rh-tube and a gas flow detector with a resolution (Mn-Kα) of 35 eV. Excitation voltage and current were 60 kV and 53 mA, respectively. The loss on ignition (LOI) was based on weight loss between 105 °C and 1000 °C and was measured after heating at 1000 °C for 2 h.

The mineralogy was analyzed by X-ray diffraction (XRD) on a X’Pert PRO diffractometer (PanAlytical, Almelo, The Netherlands), using Cu Kα radiation generated at 45 kV and 40 mA. In addition to bulk analyses, the <2 µm fraction of MS was separated by sedimentation and analyzed as orientated samples after treatments of air-drying, ethylene glycolation (vapor) at room temperature for at least 48 h, and heating at 350 °C and 550 °C for a minimum of 2 h. The mineral phases in the <2 µm fraction were identified according to [26]. The morphology of the materials (mounted on carbon tape) was investigated by scanning electron microscopy (SEM) on an FEI Quanta 200. Furthermore, total carbon (TC), sulfur (S), chloride (Cl^−^), and Atterberg limits were measured for MS. TC and S were measured using the combustion infrared detection method on LECO CS-200 (LECO Nordic, Täby, Sweden). The Cl^−^ concentration of the pore water: 4 g of dried MS was dispersed in 50 mL of distilled H_2_O for 24 h and filtrated through a 0.45 µm filter by use of vacuum pumping. Cl^−^ (mg/L) was analyzed by ion chromatography (IC). The Atterberg limits (liquid and plastic limits) were determined in accordance with DS/CEN ISO/TS 17892-12 [27]. A 0.425 mm sieve was used for the determination of the liquid limit. An additional determination of the liquid limit was carried out using the Casagrande method [28].

### 2.3. Production of Fired Specimens

To evaluate the technical properties, a small-scale test production of fired specimens was carried out at the Danish brickworks, Petersen Tegl. The raw materials were untreated prior to production; however, larger particles (>5 mm) were removed by hand if encountered during mixing and molding. Three main compositions consisting of MS (in pure form or with 10% substitutions of either CR or CH) were prepared (Table 2).

The compositions were mixed thoroughly in an industrial mixer and water was added to obtain a workable mass. The specimens were prepared by hand using the soft-mud method [29] in a water lubricated wooden mold measuring 18.6 cm × 12 cm × 6 cm (L × W × H). A wooden piston was used to aid the removal of the mold from the formed specimens. After forming, the specimens were included in the normal production routine at Petersen Tegl. They were dried at a maximum of 70 °C for 3–5 days. From each composition, half of the specimens were fired in a fully automated coal-fired tunnel kiln (reaching a maximum temperature of approximately 1045–1055 °C) and the other half were fired in an electrical furnace (reaching a maximum temperature of either 1030–1040 °C or 1070–1080 °C). In both furnaces, the heating and cooling rates were approximately 20 °C/hour, using a lowered rate of 4–10 °C/h in the interval of 520–620 °C. The specimens were maintained (soaked) at the maximum temperature for approximately 4 h.

### 2.4. Characterization of Fired Specimens

The total shrinkage of the specimens after firing was calculated as a percentage of the initial molded length before drying. Water absorption due to capillary action was determined after DS/EN 772-11 [30] at a temperature of 20 °C (±2 °C). The samples were placed in a large tray filled with distilled water to cover 5 mm of the total brick height. The weight of the samples was measured on dry-wiped samples at certain time intervals until the weight was stable for 24 h. Thereafter, the initial rate of water absorption (IRA) and capillary coefficient (k-value) were determined. For the remaining tests, the specimens were cut (parallel to the width of the specimen) into smaller pieces of approximately 4 × 10 × 5 cm (length × width × height). The open porosity and bulk density were determined according to DS/EN 772-4 [31] using a desiccator. The samples were first placed under vacuum (2 kPa) for 24 h, then under vacuum with added distilled water for 24 h, before the vacuum was released and the bricks were submerged in water for 24 h. The samples were thereafter weighed under water and immediately after being wiped dry. The compression strength was measured on an Amsler 60 tonne hydraulic press on three pieces from each specimen, which were dried at 105 °C for a minimum of three days. For comparison, two Danish red bricks, WT131 (produced by Wienerberger, Helsinge, Denmark) and D46 (produced by Petersen Tegl, Broager, Denmark), were included in the compression tests. The hygroscopic adsorption properties were determined according to ISO 12571 [32] using the desiccator method, where desiccators with different salts (LiCl, MgCl_2_, NaBr, NaCl, KCl, KNO_3_, and K_2_SO_4_) were used to keep different percentages of constant relative humidities. For this test, small samples of approximately 1 cm × 1 cm × 2 cm (L × W × H) and a weight of 3–11 g were cut from a piece from each specimen. Only one square prism from each sample was placed in the individual desiccators. The adsorption curves were established at a temperature of 20 °C (±2 °C). The bulk mineralogy of the fired specimens was investigated by XRD using the same equipment and operating conditions as described in Section 2.2.

## 3. Results

### 3.1. Raw Materials

**Figure 2:**
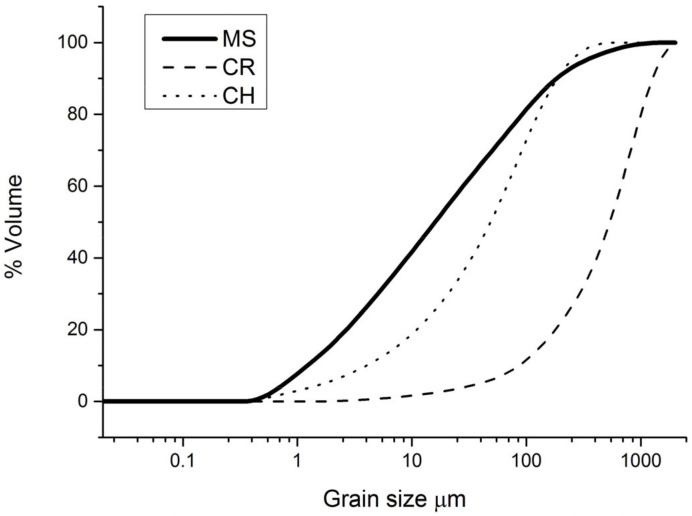
The particle size distributions of MS, CR, and CH.

The particle size distributions are presented in Figure 2. The d50 values were approximately 0.02 mm, 0.6 mm, and 0.05 mm for MS, CR, and CH, respectively. MS was dominated by the <60 µm fraction (clay and silt fractions).

**Figure 3:**
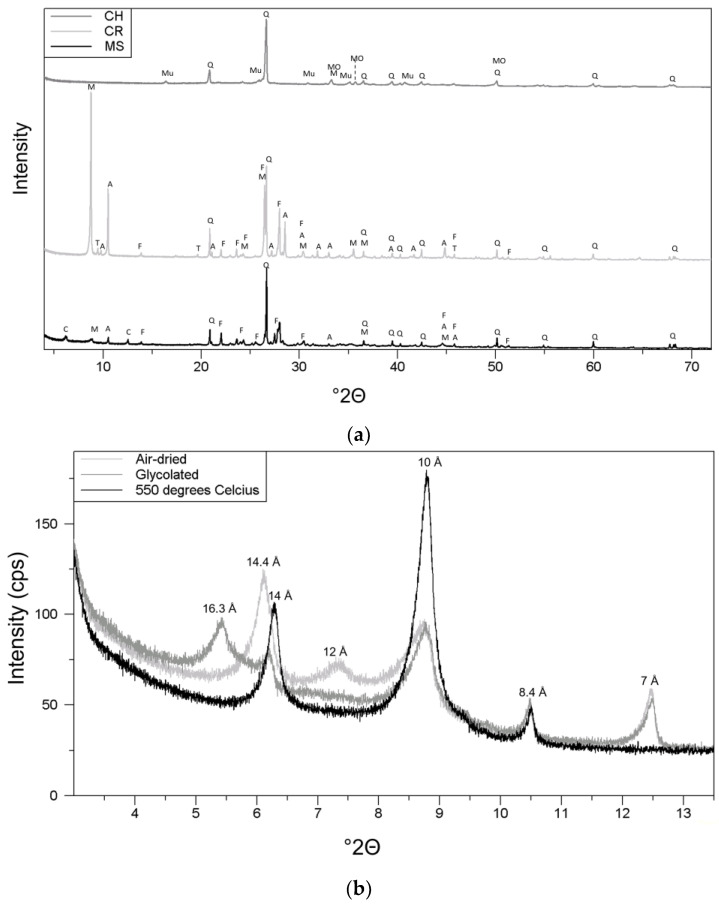
(**a**) The bulk mineralogical composition of MS, CR, and CH. A = amphibole, C = chlorite, F = feldspar, M = mica, MO = metal oxide (most likely an Fecompound, e.g., hematite), Mu = Mullite, Q = quartz, and T = talc. (**b**) The clay mineral composition of MS.

Raw material properties are presented in Figure 3 and Table 3. SiO_2_, Al_2_O_3_, and Fe_2_O_3_T were the dominating oxides for all raw materials; however, CR had a high percentage of MgO (9.8 wt%), which made MgO more dominant than Fe_2_O_3_ for this raw material. The total amounts of alkali and alkaline earth elements (K_2_O, Na_2_O, MgO, and CaO) were 12 wt%, 18.9 wt%, and 4.3 wt% for MS, CR, and CH, respectively. Additional analyses of TC, S, Cl^−^, and Atterberg limits measured for MS are also shown in Table 3.

### 3.2. Fired Brick Specimens

**Figure 4:**
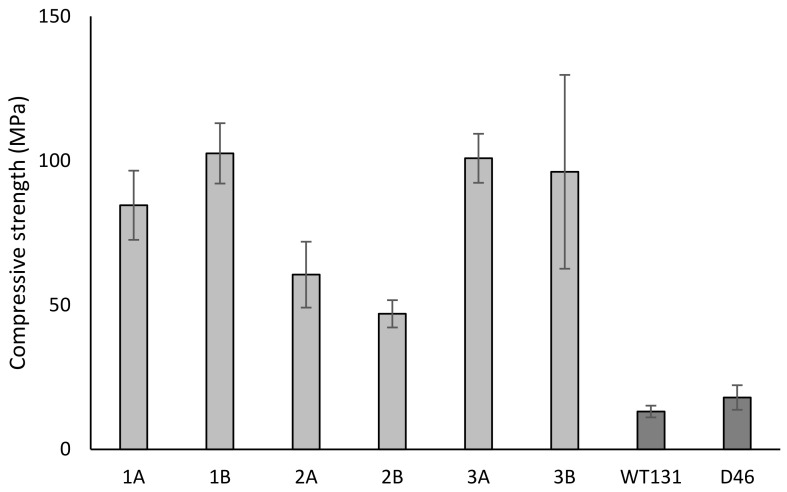
Compressive strength of bricks.

**Figure 5:**
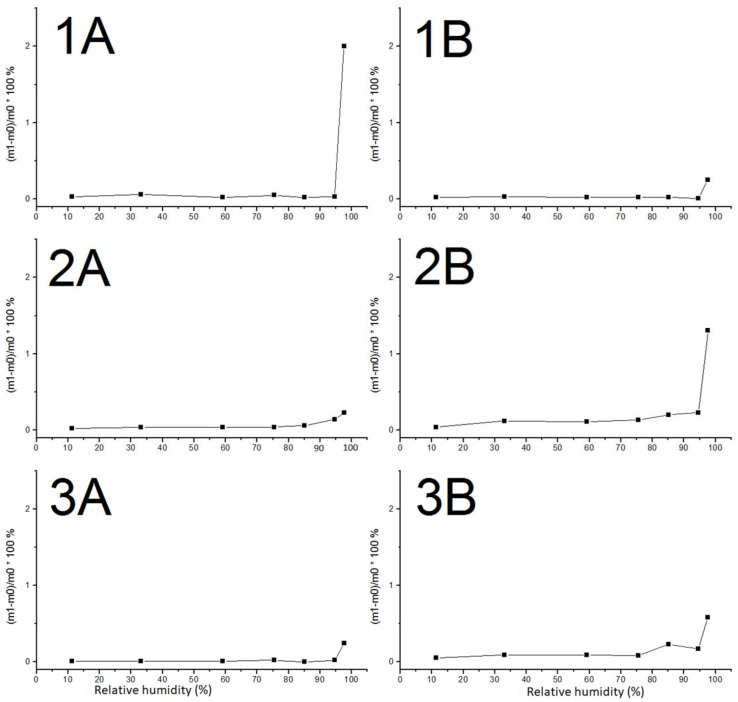
The adsorption curves of the six tested compositions (Table 2) measured at 20 ± 2 °C.

The total linear shrinkages, bulk (apparent) densities, and open porosities of the six different compositions are presented in Table 4. Composition 2B had the lowest linear shrinkage but also the highest open porosity and lowest bulk density; the opposite was true for composition 1B. The initial rate of water absorption (IRA) and capillary coefficients (k-values) are also presented in Table 4. They generally correlated well, with the IRA values being about a factor-ten higher than the k-values. The compression strengths of the six compositions and of the WT131 and D46 bricks are plotted in Figure 4, and the Greenlandic bricks showed remarkable strength, up to eight-times higher than the values obtained for the regular bricks. The adsorption curves are shown in Figure 5. The moisture content (U) is defined according to ISO 12571 [32] as (m1 − m0)/(m0) · 100%, where m0 is the initial dry mass and m1 is the mass of the same prism when equilibrium with the environment is achieved. All samples adsorbed moisture at very high relative humidity levels and only up to a few percent. As these determinations were based on the measurements of a single square prism, only the general trend can be outlined from the investigations. For compositions 2B and 3B, the adsorption initiated at a low relative humidity level, whereas it was only apparent at >80% relative humidity for compositions 1A, 1B, 2A, and 3A. 

**Figure 6:**
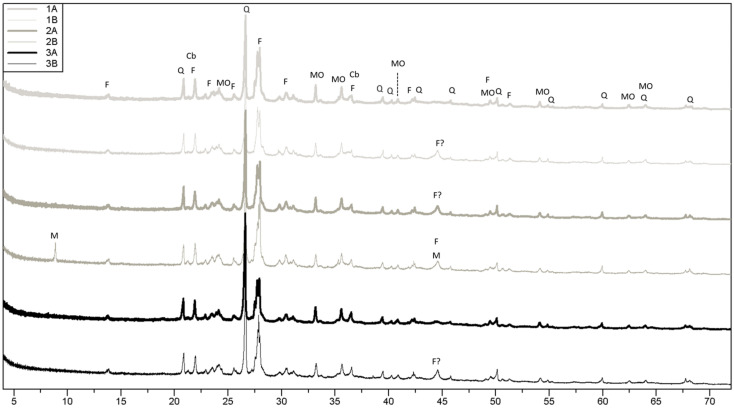
X-ray diffractograms of the fired specimens: cristobalite (Cb), feldspar (F), mica (M), metal oxide (MO), and quartz (Q).

**Figure 7:**
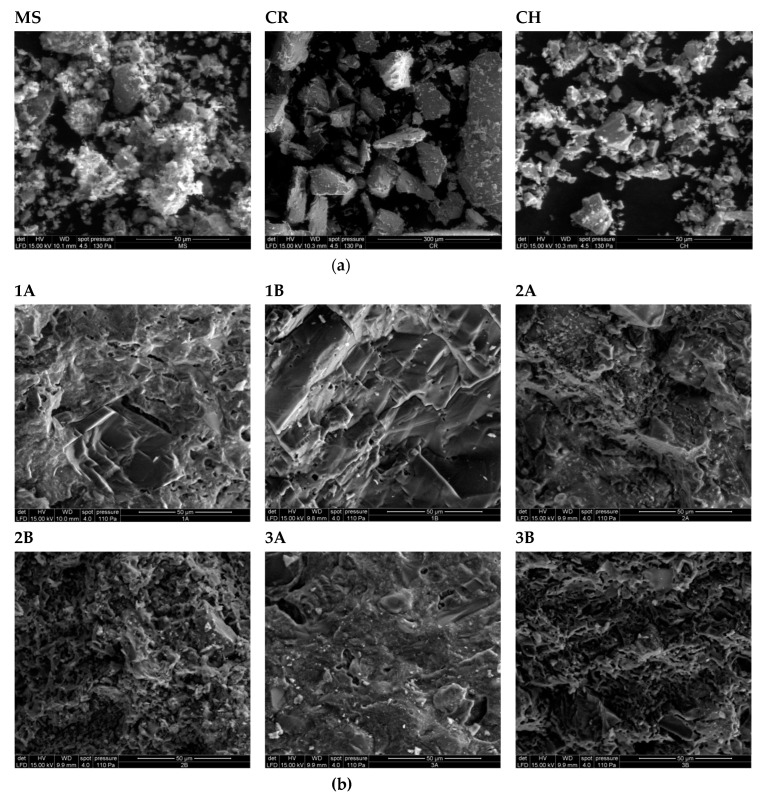
Micrographs of (**a**) raw materials (MS—marine sediment, CR—crushed rock, CH—chamotte) and (**b**) fired specimens (Table 2). The magnification of all images (bar showing 50 µm) is the same, except for CR (300 µm).

The results from the bulk mineralogy analyses of all the fired compositions are shown in Figure 6. All patterns had inherited features from MS (compared with Figure 3a). Most of the fired specimens lacked phases, such as chlorite, mica, and amphibole, in comparison to MS, but gained metal-oxide (hematite) and showed indications of the high-temperature phase, cristobalite. Peak shifts were observed for some of the feldspar peaks and were likely explained by high-temperature phase transitions, e.g., microcline to sanidine. The SEM micrographs (Figure 7) show the morphology of the raw materials and fired specimens.

## 4. Discussion

### 4.1. Raw Material Characterization

The particle size distribution, mineralogy, major element distribution, carbon content, and Atterberg limits showed that MS is very similar to other known marine clays from Greenland, Canada, and Norway [2,3,4,5,7,8]. The results from the bulk mineralogy analyses of MS, CR, and CH (Figure 3a) show the mica in MS to be dominantly trioctahedral (biotite-type), based on a (060) reflection of around 1.54 Å and very weak peaks in the 1.50 Å region [22]. For CR, the octahedral coordination was less clear, but appeared to be more trioctahedral based on stronger reflections around 1.54 Å than around 1.50 Å. The high MgO wt% observed for CR (Table 3) corresponds well with the abundant mica (biotite) found from the XRD investigations. For CR, the presence of pyroxene was also indicated; however, due to peak overlapping, this could not be clearly resolved. For CH, cristobalite was observed, but is not displayed in the figure due to the low intensity of the peaks. The result of the clay mineral analyses of MS is presented in Figure 3b. Expandable (smectitic) clay was identified based on the glycolated peak around 16.3 Å. Mixed-layer clay was identified based on the air-dried peak around 12 Å, which disappeared after glycolation and chlorite was identified based on the presence and increase in the 14 Å peak and the disappearance of the second-order (7 Å) reflection at 550 °C. The shift from 14.4 Å to 14 Å from air-dried to 550 °C could indicate the presence of vermiculite. Illite/biotite was identified from the 10 Å peak (before heating). The 8.4 Å peak (Figure 3b) was assigned to amphibole, which together with quartz and feldspar peaks from the bulk analysis (Figure 3a) confirmed the presence of primary minerals in the <2 µm fraction.

The low TC (Table 3) was consistent with the low content of organic carbon observed for other marine clays in Greenland, Canada, and Norway (e.g., [1]). Furthermore, the low TC also indicated a low content of CaCO_3_, which, combined with the relatively high amount of Fe_2_O_3_T, contributed to the red coloration of the bricks after firing. The low chloride concentration of MS indicates that, in spite of its marine origin, the original salt content in the pore water has since been leached out. The morphology of the three raw materials as studied by SEM revealed that CR and CH generally contained grains of euhedral textures, whereas the grains in MS had a more subhedral texture. Furthermore, the surfaces of larger grains in MS were generally covered by smaller grains with a flaky or powdery appearance, which were likely to be weathering products, e.g., clay minerals. This observation was less pronounced for CR and CH.

The relatively low plasticity index of MS (Table 3) presents a challenge when producing bricks by the soft-mud method. The extrusion (stiff mud) or dry-pressing methods [29] would therefore possibly be better for industrial production using this type of clay.

### 4.2. Influence of Production Settings

To observe the effect of the different temperatures on the technical properties of the produced bricks, the pure MS compositions, 1A and 1B, were fired initially at 1045–1055 °C and 1070–1080 °C, respectively. The higher firing temperature of 1B led to an increase in total shrinkage, bulk density, and compression strength, and a decrease in water absorption and open porosity. As no obvious benefits could justify the higher temperature and therefore energy consumption, it was decided to use lower firing temperatures of 1030–1055 °C for compositions 2A, 2B, 3A, and 3B. Furthermore, the relatively high total shrinkage of both 1A and 1B gave rise to the idea of introducing the two additives, CR and CH, both of which had a coarser grain size distribution and a lower LOI than MS and therefore were less likely to contribute to shrinkage.

Compared to small brick discs produced at the laboratory scale, which were formed and pressed at 47 MPa, the open porosity (39.5%), density (1691 kg/m³), and water absorption (23.3%) [15] were significantly different from the bricks made at the brickwork in this study, even when using the same marine sediment. Additionally, the firing curve in the laboratory was faster and performed at a lower temperature (maximum 1000 °C for 1 h [10]). These findings emphasis the importance of developing laboratory production methods that closely correlate with industrial production, both in terms of forming pressure and firing curve, to enable direct comparisons of brick properties between laboratory and pilot scales.

### 4.3. Shrinkage

As expected, the total shrinkage reduced for the CR-containing composition (2A) compared to the pure MS composition (1A). However, despite a coarser grain size distribution of CH compared to MS (Figure 2), there was no observable difference in total shrinkage between compositions 3A and 1A. Lowering the firing temperature to 1030–1040 °C for compositions 2B and 3B also reduced the total shrinkage. According to the Brick Industry Association [29], the total size shrinkage after drying and firing can vary for different clays, but will usually be around 4.5–8%. For the compositions tested here, only 2B falls within the higher end of this range. However, although the total shrinkage is generally in the range of 11–15% for the compositions in this study, it does not appear to be detrimental, as only very few cracks were observed on the surfaces of the specimens after firing. Most of these cracks were correlated to underlying larger rock fragments and could possibly have been avoided by crushing or by particle size sorting of the material before production, which is standard practice in industrial brick manufacturing.

### 4.4. Initial Rate of Water Absorption and Hygroscopic Behavior

According to ASTM C216-14 [33], the initial rate of water absorption (IRA) for facing bricks should be up to 1.5 kg/(m^2^∙min). Bricks with higher IRA values require pre-wetting, as they will otherwise prevent the complete hydration of the cement, due to the absorption of water from mortar. Bricks with a low IRA should be covered on the construction site in order to prevent wetting, as this might prevent or delay the formation of bonds between the brick and the cement. The compositions 1A, 1B, 2A, and 3A, which were fired at temperatures of 1045–1080 °C, all have low IRA values, whereas compositions 2B and 3B, which were fired at 1030–1040 °C, have IRA values closer to 1.5 (Table 4). All compositions had very low hygroscopic adsorption (Figure 5). However, the adsorption of water at lower relative moisture contents for compositions 2B and 3B compared to the other compositions indicates that 2B and 3B have larger available inner surface areas where water can adsorb. Furthermore, the combined studies on hygroscopic adsorption and capillary uptake (Table 4) indicate that the higher firing temperatures of compositions 1A, 1B, 2A, and 3A reduced the amount of pores capable of capillary uptake compared to the lower-temperature compositions 2B and 3B.

### 4.5. Degree of Sintering

Vieira and Monteiro [34] investigated a fine-grained granitic waste material for incorporation in ceramics, which had a d50 of approximately 10 µm. They concluded that the fine particle size distribution favored sintering and reduced the porosity in the produced ceramics. Furthermore, the material was shown to have a considerable fluxing potential at temperatures around or slightly above 1000 °C due to a high amount of alkaline oxides present in the form of biotite and Na,K-feldspar, which contributed to the formation of a liquid phase. As both the grain size distribution and qualitative mineralogy of the material characterized by Vieira and Monteiro [34] are comparable to MS, it is likely that similar reactions would occur here. In addition, the very fine particle sizes observed on the surfaces of the grains in MS (Figure 7) are also likely to enhance sintering. When comparing the XRD results of the raw materials to the XRD results of the fired compositions, it is evident that phases such as mica (biotite), amphibole, and chlorite disappeared after firing, which could be explained by the breakdown/melting of these phases at or below the temperatures investigated. Interestingly, composition 2B, which was fired at 1030–1040 °C, still contains some mica (peak at approximately 8.9°2Θ), which is not evident in composition 2A fired at 1045–1055 °C. This indicates that at least some of the mica encountered in CR is stable at temperatures above the range of 1030–1040 °C, but will breakdown at or below temperatures of 1045–1055 °C. The mica in MS appears to breakdown at temperatures below the range of 1030–1040 °C as no mica peaks are apparent in composition 3B, which was fired at this temperature.

The coarser particle size distribution of CR, which has a d50 of approximately 600 µm, is less likely to enhance sintering and reduce porosity, which is consistent with the reduced strength (Figure 4) and higher open porosity values found for composition 2A compared to 1A (Table 4). In general, the strength, density, and shrinkage of most of the tested compositions indicate a high degree of sintering.

## 5. Conclusions

The results demonstrate that the marine sediment from Ilulissat (MS) is suitable as brick clay and that it is possible to produce high-quality bricks. The produced specimens were fired at temperatures in the range of 1030–1080 °C. The highest temperatures did not contribute to further enhancements of the technical properties, and the temperature range of 1030–1055 °C was therefore sufficient for the marine sediment. The specimens fired at 1045–1055 °C generally had very high compression strengths, low porosities, and low water absorptions. The introduction of 10% of the crushing rock residue gave rise to a reduction in strength, higher porosity, and water absorption. In addition, the specimens fired at 1030–1040 °C had the lowest strength, highest porosity, and water absorption. On the contrary, the use of 10% chamotte with marine sediment did not impair the bricks’ properties. The bricks showed greater shrinkage compared to previous, small laboratory samples, and show the importance of conducting large-scale tests under industrial forming pressures and firing conditions.

## Figures and Tables

**Table 1:** Major element composition of marine sediments and brick clays.

	Glaciogene Marine Sediments					
Chemical Oxide (wt%)	Greenland (North Atlantic Craton) [9]	Greenland (Nagssugtoqidian Orogen) [10,11,12]	Norway [5,8]	Canada [2,3]	Marine Sediments Used for Clay Bricks [13,14,15,16]	Typical Clay for Danish Red Bricks [17]
SiO_2_	53.99	57.5–64.2	45.8–53.6	46.8–62.6	47.13–63	55.46–76.97
TiO_2_	0.71	0.6–0.8	0.8–1.1	0.0–1.0	0.63–0.81	
Al_2_O_3_	16.44	14.5–16.2	14.3–21.5	15.4–23.4	7.69–17.23	9.59–14.41
Fe_2_O_3_T	8.55	6.2–6.6	8.2–15.2	4.4–8.8	4.13–21.55	3.76–6.96
MnO	0.12	0.1	0.0–0.2	0.1	0.03–0.31	
MgO	4.94	2.4–3.8	3.9–5.1	2.1–4.9	0.05–3.6	0.87–1.71
CaO	3.72	2.4–4.4	1.0–4.1	2.9–5.4	0.68–7.49	0.68–2.48
Na_2_O	3.84	3.3–4.5	1.2–2.4	1.3–4.1	0.61–1.9	0.48–1.19
K_2_O	3.5	2.7–2.9	4.7–6.0	2.4–3.7	1.36–2.13	2.49–3.0
P_2_O_5_	0.12	0.1–0.2		0.1–0.3	0.15–0.32	0.06–0.12
LOI	3.72	2.4–5.1	3.5–10.3	1.6–11.9	10–19.35	

**Table 2:** Specifications for the test specimens.

Specimen Name	MS (wt%)	CR (wt%)	CH (wt%)	No. of Specimens	Coal Furnace	Electrical Furnace	Firing Temperature (°C)
1A	100			2	X		1045–1055
1B	100			3		X	1070–1080
2A	90	10		3	X		1045–1055
2B	90	10		2		X	1030–1040
3A	90		10	3	X		1045–1055
3B	90		10	3		X	1030–1040

**Table 3:** Characteristics of raw materials. N.M. = not measured.

		MS	CR	CH
Major elements (XRF)	SiO_2_ (wt%)	61.12	61.77	66.94
	TiO_2_ (wt%)	0.59	0.39	1.28
	Al_2_O_3_ (wt%)	14.52	10.63	19.22
	Fe_2_O_3_T (wt%)	6.48	6.45	7.80
	MnO (wt%)	0.08	0.11	0.06
	MgO (wt%)	3.52	9.67	0.99
	CaO (wt%)	2.36	3.64	0.33
	Na_2_O (wt%)	3.31	3.11	0.43
	K_2_O (wt%)	2.81	2.51	2.59
	P_2_O_5_ (wt%)	0.12	0.04	0.06
	LOI_1000 °C_ (wt%)	5.09	1.68	0.30
	TC (wt%)	1.41 ± 0.18	N.M.	N.M.
	S (wt%)	0.10 ± 0.08	N.M.	N.M.
	Cl^−^ (mg/L)	3.29 ± 0.04	N.M.	N.M.
Atterberg limits	Water content (%)	23.5 ± 1.7	N.M.	N.M.
	Liquid Limit (%)	30.2 ± 0.2	N.M.	N.M.
	Plastic Limit (%)	20.5	N.M.	N.M.
	Plasticity Index	9.7	N.M.	N.M.
	Activity	0.23	N.M.	N.M.

**Table 4:** Properties of brick specimens.

	Total Shrinkage %	Bulk (Apparent) Density kg/m^3^	Open Porosity %	IRA $kg/(m^{2}$× min)	k-Value $kg/(m^{2}$$\times \;s^{½})$
1A	13.8 ± 0.5	2350	4.9	0.170 ± 0.060	0.018 ± 0.005
1B	14.9 ± 0.4	2400	2.9	0.080 ± 0.050	0.008 ± 0.004
2A	11.0 ± 0.5	2260	16.8	0.300 ± 0.010	0.035 ± 0.003
2B	7.7 ± 0.8	1960	38.2	1.630 ± 0.060	0.183 ± 0.010
3A	13.8 ± 0.2	2360	9.3	0.080 ± 0.020	0.009 ± 0.003
3B	11.0 ± 0.4	2090	27.2	0.890 ± 0.180	0.094 ± 0.019

## Data Availability

The raw data supporting the conclusions of this article will be made available by the authors on request.
